# Tobacco Screening Practices and Perceived Barriers to Offering Tobacco Cessation Services among Texas Health Care Centers Providing Behavioral Health Treatment

**DOI:** 10.3390/ijerph19159647

**Published:** 2022-08-05

**Authors:** Ammar D. Siddiqi, Maggie Britton, Tzuan A. Chen, Brian J. Carter, Carol Wang, Isabel Martinez Leal, Anastasia Rogova, Bryce Kyburz, Teresa Williams, Mayuri Patel, Lorraine R. Reitzel

**Affiliations:** 1 Department of Biosciences, Rice University, 6100 Main St., Houston, TX 77005, USA; 2 Department of Psychological, Health & Learning Sciences, The University of Houston, 3657 Cullen Blvd Stephen Power Farish Hall, Houston, TX 77204, USA; 3 Health Research Institute, The University of Houston, 4349 Martin Luther King Blvd, Houston, TX 77204, USA; 4 Department of Health Disparities Research, MD Anderson Cancer Center, Houston, TX 77230, USA; 5 Integral Care, 1430 Collier St., Austin, TX 78704, USA; 6 Department of State Health Services, Tobacco Prevention and Control Branch, Austin, TX 78714, USA

**Keywords:** tobacco screening, screening practices, behavioral populations, health disparities, Texas, mental illness, substance abuse, cessation care, perceived barriers, facilitators

## Abstract

Tobacco use, and thus tobacco-related morbidity, is elevated amongst patients with behavioral health treatment needs. Consequently, it is important that centers providing health care to this group mandate providers’ use of tobacco screenings to inform the need for tobacco use disorder intervention. This study examined the prevalence of mandated tobacco screenings in 80 centers providing health care to Texans with behavioral health needs, examined key factors that could enhance screening conduct, and delineated providers’ perceived barriers to tobacco use intervention provision. The results indicated that 80% of surveyed centers mandated tobacco use screenings; those that did were significantly more likely than those that did not to have a hard stop for tobacco use status in health records and were marginally more likely to make training on tobacco screening available to providers. The most widespread barriers to tobacco use disorder care provision were relative perceived importance of competing diagnoses, lack of community resources to refer patients, perceived lack of time, lack of provider knowledge or confidence, and belief that patients do not comply with cessation treatment. Overall, the results suggest that there are opportunities for centers providing care to Texans with behavioral health needs to bolster their tobacco screening and intervention capacity to better address tobacco-related health disparities in this group. Health care centers can support their providers to intervene in tobacco use by mandating screenings, streamlining clinical workflows with hard stops in patient records, and educating providers about the importance of treating tobacco with brief evidence-based intervention strategies while providing accurate information about patients’ interest in quitting and providers’ potential impacts on a successful quit attempt.

## 1. Introduction

As a result of being the leading cause of preventable illness and death, tobacco use in the US remains a persistent problem [1,2,3,4]. Tobacco product use has been causally linked to illnesses including but not limited to cancer, cardiovascular disease, stroke, and diabetes. Despite this, nearly 40 million US adults still utilize tobacco products [1]. Although the prevalence of smoking has declined over the past forty years in the general population, tobacco use amongst some population subgroups has followed a different trajectory, remaining an issue that has yet to be adequately addressed [5,6]. Of those who have been disparately impacted, national public health organizations have adopted the term behavioral health to refer to those suffering from substance abuse or mental health-related illnesses [7]. Multiple studies have demonstrated that individuals in this category face greater tobacco-related morbidity from their elevated tobacco use, cited as about two to four times greater than the general US population. As a result, individuals with behavioral health needs are a tobacco-related health disparity group despite not being ubiquitously recognized as one [5,6,8,9,10,11,12,13,14]. Thus, there is a dire need to address tobacco use among behavioral health patients.

The integration of tobacco screenings into health care settings is an evidence-based method of facilitating tobacco cessation among diverse patient groups [15,16,17,18,19,20]. Moreover, comprehensive tobacco screenings, according to The National Commission on Prevention Priorities, have the most clinically favorable outcomes for patients and are more cost-effective than other preventive interventions for common health conditions such as hypertension and high cholesterol [15,17]. Furthermore, comprehensive tobacco use screenings are a natural first step to providing cessation services, especially important for clinical encounters with tobacco disparity groups that may be less likely to receive tobacco use disorder care [11,21,22,23,24]. Consequently, the Affordable Care Act designates preventive services encompassing tobacco screenings as one of ten essential health benefits that are required to be covered by private insurance and most state Medicaid programs [25,26]. However, despite the widespread evidence for the significance and feasibility of conducting tobacco use screenings in health care settings, several studies have suggested limitations in their uptake [8,23,27,28,29]. Specifically, tobacco screenings are reported to occur with between 37.9% and 70% of patients, whereas national recommendations suggest that all adult patients should be assessed in every clinical encounter, with comprehensive tobacco use assessments (TUAs) delivered to those endorsing consumption to guide cessation interventions [22,28,30,31,32,33,34]. Unfortunately, behavioral health patients, compared with the general population, may be disproportionately less likely to receive tobacco use screenings in settings where they receive care, thereby perseverating the tobacco-related health disparities within this group [8,11,27,28,29,30,35].

The lack of consistent tobacco screening provision in health care settings has been attributed to limited knowledge and training of treatment providers [36,37,38,39,40,41]. For example, a recent study reported that 90% of surveyed health care providers did not have recent tobacco counseling training within the last 12 months, and 73.3% of them were unaware of evidence-based brief interventions for tobacco dependence that included asking patients about their use [36]. Limited training and a lack of standardized screening procedures for tobacco use disorder have significant impacts on intervention provision, as can the perception of limited time to provide preventive screening services if not related to the patient’s chief complaint [31,40,41,42,43]. Research suggests that integrating tobacco screenings into standardized clinical procedures encourages routine screening and intervention; an example of such practice is the implementation of hard-stop alerts in patients’ electronic health records (EHR), which prevents clinicians from further modifying the EHR without an entry about tobacco use status or a manual override [24,36,37,44,45]. However, hard stops are not ubiquitous within EHR systems, and some settings where tobacco-using patients are routinely seen are still utilizing paper charts for clinical encounters [46,47]. This may especially be the case in states like Texas where, according to data collected in 2016, only 58.4% of mental health treatment centers and 70.2% of substance use treatment centers were screening patients for tobacco use [24].

There remains limited understanding regarding how factors affecting screening practices create barriers to tobacco use care provision within settings where behavioral health patients receive care [7]. Texas, the second-largest state in the US, is known for its rapid growth and diverse demographics, has six of the twenty-five largest US urban cities, and has the greatest number of residents living in rural areas (where tobacco use rates are higher than in urban areas), making it an important setting in which to explore tobacco use screening practices [48,49]. Additionally, given known statewide efforts to enhance tobacco use disorder care delivery in Texas since 2016 [24,50,51,52,53], more recent data are needed to understand any changes in tobacco use screening practices within behavioral health care settings since the prior report. Finally, the exploration of tobacco screening practices and associated limiting factors for behavioral health patients should expand beyond traditional treatment settings (e.g., mental health centers). This is because patients may receive behavioral health care in primary care settings, particularly in rural areas of the state where behavioral health treatment centers are less prevalent [54,55]. The current study was conducted to address these gaps. Understanding more about factors that can act as barriers to or facilitators of tobacco screening and care in diverse treatment settings can further contextualize observed trends and, in turn, provide avenues for future improvement to reduce tobacco-related disparities in behavioral health groups.

## 2. Materials and Methods

### 2.1. Targeted Health Care Centers

Data were collected as part of a contracted statewide needs assessment with the purpose of better understanding current policies and practices in addressing patients’ tobacco use within diverse treatment settings providing behavioral health care across the state of Texas. These health care centers included Federally Qualified Health Centers (FQHCs), which are important sources of sliding fee-scale care provided within community settings [56] that can include behavioral health care for low-income patients. Another targeted setting was state-supported Local Mental Health Authorities (LMHAs), which are located across Texas and provide low-cost behavioral health care services to patients across a range of (primarily mental health) diagnoses [57]. A third health care center target was substance use treatment programs within LMHAs, which although encapsulated within global LMHAs are very specific to the treatment of substance use disorders and consequently operate somewhat independently from them. A final health care target was standalone substance use treatment programs, which can provide either broad or specific (e.g., opioid dependence care) substance use care.

### 2.2. Survey Methods and Response Rates

Data collection spanned April 2021–December 2021. Although the study did not meet the definition of human subject research per the University of Houston’s IRB determination, a cover letter describing the purpose of the study and including essential elements of informed consent preceded an electronic Qualtrics survey. This cover letter indicated that respondents could receive a $20 Amazon gift card for completing at least 75% of the survey.

The study team, along with community liaisons from the HEALTH Research Institute at the University of Houston, collected contact information for health care centers from various online sources [58]. The recruitment of centers included direct email solicitations to this list and/or postal mail solicitations to those centers where a contact email was not evident. Additional recruitment strategies included team attendance as a vendor at professional organization meetings/conferences for health care center stakeholders, where the needs assessment was promoted with representatives available to answer any questions. The team also obtained recruitment assistance from professional organization leadership who were willing to send us their member lists or to send information about the needs assessment through their listservs. Finally, the team worked with the Texas Department of State Health Services, the contractor of the needs assessment, to promote the study in conjunction with the delivery of tobacco treatment education within regular meetings of the Tobacco Prevention and Control Coalitions, regional coordinator meetings, and other similar meetings (e.g., Community Resource Coordinating Groups).

The study team solicited participation from Texas FQHCs (*n* = 57), all global LMHAs (*n* = 39), identified substance use treatment programs in LMHAs (*n* = 89), and identified standalone substance use treatment centers (*n* = 458). Overall, the goal was to receive one completed or near-completed survey per physical location of the health care center target; this was difficult to control through our survey solicitation efforts, so in some cases, there were duplicates. Duplicates (*n* = 10, representing 5 centers) were handled in a stepwise manner. First, completed surveys were kept over partially completed surveys. Next, responses from direct service providers were maintained over general employees who had no patient contact to ensure the maximum retention of data (direct service providers completed additional questions relative to their counterparts) and because we believed they would be relatively more aware of how tobacco use was being handled at their center. Finally, we elected to retain surveys of the respondents most likely to be informed about tobacco-related topics based on their job title (e.g., an executive director over an administrative assistant). This strategy yielded the following representation: 43.9% of solicited FQHCs (*n* = 25/57), 76.9% of global LMHAs (*n* = 30/39), 15.7% of substance use treatment programs within LMHAs (*n* = 14/89), and 14.4% of standalone substance use treatment centers (*n* = 66/458). Because the present report is focused on questions only asked of direct services providers, the analyzable sample was further narrowed to 9 FQHCs, 16 global LMHAs, 6 substance use treatment programs in LMHAs, and 55 standalone substance use treatment centers. 

### 2.3. Measures

#### 2.3.1. Health Care Center Characteristics

Descriptive health care center characteristics assessed were: (1) the number of unique patients seen annually (later categorized based on sample distribution as 50–200; 201–1000; or >1000); (2) the number of full-time employees (later dichotomized based on sample distribution as 1–50 vs. >50); (3) whether the center employed a person trained as a Certified Tobacco Treatment Specialist (CTTS; yes vs. no/I don’t know); and (4) whether the center had a comprehensive tobacco-free workplace policy, defined as having endorsed that tobacco use was disallowed indoors and on the center’s property (endorsed vs. not endorsed).

#### 2.3.2. Health Care Center Tobacco Screening Practices/Resources and Perceived Barriers

Survey respondents answered items regarding their health care center’s (i.e., not their own) tobacco screening practices, which yielded the independent and dependent variables of interest for this report. The dependent variable was whether the center mandated that every adult patient be screened for tobacco use at intake and that this be documented in the patient record (yes vs. no/I do not know). The independent variables were whether the center: (1) provided a template or guide for a TUA that included information like the patient’s smoking status, smoking history, cigarettes or packs smoked per day, number of years smoking, number of years since quitting smoking, etc. (yes vs. no/I do not know); (2) used an EHR with a hard stop that required the entry information about patient tobacco use status (yes vs. no/I do not know or N/A, we do not have an EHR); and (3) received or offered training to direct service providers on how to screen patients for tobacco use (yes vs. no/I do not know).

Respondents also answered an item regarding recognized factors limiting their health care center’s (not their own) provision of tobacco screening and intervention, whereby any applicable barrier could be endorsed on a 5-point Likert scale of importance that ranged from not at all important to extremely important or identified as not applicable (N/A). Options included: (1) patients are not interested; (2) patients do not comply with treatment; (3) organizational leadership is not interested/invested in this; (4) employees are not interested/invested in doing this; (5) employees do not know how to do this or lack confidence in their abilities to do this; (6) lack of impact on patients; (7) lack of time for direct service providers to provide tobacco cessation services; (8) lack of reimbursement/it costs too much to do this; (9) lack of community resources to refer patients; (10) lack of patient education material; (11) lack of training; (12) complexity of smoking or other tobacco use cessation guidelines; and (13) relative importance of competing problems/diagnoses/comorbidities. For analytic purposes, responses were grouped as follows: not at all important/slightly important/moderately important or N/A (i.e., not endorsed as a barrier) vs. very important or extremely important (i.e., endorsed as a barrier).

### 2.4. Data Analyses

Of the 86 respondents, we excluded 6 providers who had incomplete information on the key variables of interest, resulting in a final analytic sample of 80. Data on health care center characteristics and mandated tobacco use screening were reported with descriptive statistics. Comparisons between center types on these variables were assessed using chi-square tests. Next, the limiting factors to offering tobacco use intervention, including screening, to patients were explored, whereby key factors were defined as those endorsed by at least 50% of the respondents within each health care center type. The most widespread barriers in the sample overall were defined as those reported by at least 50% of all respondents at ≥1 type of health care center. Then, three logistic regression analyses were conducted to assess the associations between each of the independent variables (key factors to enhance tobacco screening: TUA template availability, EHR hard stop/documentation required, and training to screen) and the dependent variable, center-mandated tobacco use screening. Health care center type and comprehensive tobacco-free workplace policy presence were included as covariates in the analyses to reduce potential confounding effects. The main analysis included all independent variables in a single model with covariates to assess the extent to which each contributed unique variance in its association with mandated tobacco screening. Statistical significance was designated at *p* < 0.05, with values between *p* = 0.05 and 0.10 considered marginally significant. All analyses were conducted using SAS version 9.4.

## 3. Results

### 3.1. Healthcare Center Characteristics and Screening Mandates

Of the 80 centers included in the analyses, 44.87% (*n* = 35) reported serving 201–1000 unique patients yearly, 61.25% (*n* = 49) reported having 1–50 full-time employees, 30% (*n* = 24) had at least one CTTS, 50% (*n* = 40) had a comprehensive tobacco-free workplace policy, and 80% (*n* = 64) mandated tobacco use screening. There were significant center type differences on number of unique patients served yearly (*X*^2^ = 38.701, *p* < 0.001) and number of full-time employees (*X*^2^ = 34.254, *p* < 0.001). Global LMHAs tended to serve more unique patients yearly and had more full-time employees than other center types (Table 1).

### 3.2. Perceived Health Care Center Barriers to Tobacco Use Disorder Care

Key barriers to providing tobacco use disorder screening and intervention were defined as those endorsed by at least 50% of the sample within each respective health care center type. The responding FQHCs endorsed four key barriers: (1) relative importance of competing diagnoses (66.7%); (2) lack of time (66.7%); (3) patients are not interested (55.6%); and (4) patients do not comply with treatment (55.6%). In the global LMHAs, the three key barriers were: (1) relative importance of competing diagnoses (68.8%); (2) lack of time (62.5%); and (3) lack of provider knowledge or confidence (53.3%). The responding substance use treatment programs in LMHAs endorsed only one key barrier: the lack of community resources to refer patients (60.0%). In the standalone substance use treatment centers, eight key barriers were endorsed: (1) lack of training (67.4%); (2) lack of community resources to refer patients (62.5%); (3) lack of patient education material (58.3%); (4) lack of time (58.3%); (5) relative importance of competing diagnoses (55.1%); (6) lack of provider knowledge or confidence (54.2%); (7) lack of provider interest (50.0%); and (8) patients do not comply with treatment (50.0%) (data not presented in tabular form). 

The most widespread barriers in the sample overall were defined as those reported by at least 50% of providers at ≥1 type of health care center. These widespread barriers were: (1) the relative perceived importance of competing diagnoses (key barrier for three center types), (2) perceived lack of time (key barrier for three center types), (3) lack of community resources to refer patients (key barriers for two center types), (4) patients do not comply with treatment to quit (key barriers for two center types), and (5) a lack of provider knowledge or confidence (key barriers for two center types).

### 3.3. Health Care Center Tobacco Use Screening Mandates in Relation to Practices/Resources

In the series of three adjusted logistic regressions predicting mandated tobacco screening, significant associations were found for centers that used an EHR with a hard stop for patient tobacco use status (*p* < 0.008) and those providing or receiving training on screening for tobacco use (*p* = 0.016). The availability of a template for TUAs was not associated with mandated tobacco use screenings (*p* = 0.153). The main analysis with all independent variables entered conjointly and adjusted for health care center type and presence of a comprehensive tobacco-free workplace policy revealed that having an EHR with a hard stop for tobacco use status entry maintained statistical significance in its association with mandated tobacco use screening, whereas the provision or receipt of training on screening became marginally significant (Table 2). 

## 4. Discussion

Tobacco disparity groups, including those suffering from mental illnesses and substance use disorders, utilize tobacco products at a disproportionate rate and face greater tobacco-related morbidity as a result [5,6,8,9,10,11,12,13,14]. The disproportionate use of tobacco can effectively be addressed by increasing the occurrence of tobacco screenings, a cost-effective method for improving patient outcomes in settings where patients with behavioral health disorders receive care [15,17]. Results from this Texas-based study demonstrated that only 80% of participating health care centers endorsed mandating tobacco screenings of their patients, clearly suggesting opportunities for 20% of centers to improve their policies and practices in this regard. Moreover, there were no significant differences between the health care centers surveyed (i.e., FQHCs, global LMHAs, substance use programs in LMHAs, and standalone substance use treatment centers) in mandating tobacco screening, suggesting opportunities for improvement in every practice setting where Texans may receive behavioral health care. These findings, unfortunately, are consistent with previous reports showing that tobacco screenings are not a ubiquitous practice within different health care settings, despite national treatment guidelines underscoring their importance [24,30,31,32,33,34,35]. However, although it is highly likely that these studies had different survey respondents, the results indicate that rates of tobacco screening in Texas’ mental health and substance abuse treatment centers have increased over time (i.e., from 58.4% and 70.2%, respectively, in 2016 [24] to 81.25% and 80%, respectively, in the current 2021 data collection). This could be the result of several known public health efforts in Texas geared toward improving tobacco use disorder care in behavioral health treatment settings (e.g., [50,51,52,53,59,60,61,62,63,64,65,66,67,68]). Regardless, tobacco screening should be occurring with 100% of adult patients in these settings, as well as in other health care settings where patients with behavioral health care needs are seen (like FQHCs), indicating the need for more progress in this area. Further gains might be achieved by making health care centers’ receipt of state and federal funding contingent upon a contractual requirement to provide tobacco use screenings to all adult patients at each health care contact. Optional recommendations to screen patients for tobacco use as posited by accredited health care oversight organizations, or mandates without an accountability process in place, may have limited effects on those health care centers that have lagged in their adoption of evidence-based tobacco use care disorder policies and practices [34,69].

Common barriers to tobacco interventions, including screening for tobacco use, in clinical settings have been reported in previous literature but were studied in greater detail within this study across different types of health care centers. Although the key barriers differed by setting, the most widespread barriers reported by providers were relative perceived importance of competing diagnoses, lack of community resources, lack of time, lack of knowledge or confidence, and belief that patients do not comply with treatment to quit. These barriers do not appear to be unique to providers of behavioral health care but may be reported in greater frequency within these settings [36,37,38,39,40,41]. Moreover, previous research has reinforced that many of these perceived barriers are just that, perceptions that can be overcome through training and education. For example, brief tobacco training as short as one hour has been effective in increasing the relevance of intervention by equipping health care personnel with pertinent knowledge and strategies to address the sheer morbidity associated with tobacco use—projected to kill over a billion in the next century [39,70]. Brief tobacco intervention practices, such as the administration of the 5A’s (ask patients about their tobacco use, advise them to quit, assess their willingness to quit, assist them in quitting, and arrange for follow-up), can accommodate the time constraints faced by providers by requiring as little time as a minute to administer to encourage tobacco cessation [34,71,72]. These practices can be complemented with referral to quitline services, which have been shown to significantly increase the probability of a successful quit attempt [73]. Despite being a free and accessible community resource to help coach callers on how to stop using tobacco products, quitlines are only utilized by 1% of tobacco users in the US [74]. Therefore, to increase the visibility of quitlines amongst providers and their use amongst patients, greater dissemination of information on the services they offer is needed. Additionally, some reported provider barriers were rooted in assumptions about patients’ motivations. These assumptions can affect providers’ provision of indispensable support for cessation and can be addressed through education [75,76]. Specifically, the sentiment that patients do not follow provider treatment advisement was a commonly held provider belief that is not reflective of most behavioral health patients. In fact, patients with behavioral health needs attempt to quit using tobacco products at higher rates than the general population due to the disproportionate impact tobacco products have on their health and the noticeable benefits quitting can have on their well-being like improved pulmonary function [77,78]. Furthermore, struggling to quit products containing nicotine, a highly addictive substance found in tobacco products, is common, taking on average 30 attempts before quitting, with only 7.5% of users succeeding in achieving prolonged abstinence annually [79,80]. The difficulty of tobacco cessation and the unique characteristics of behavioral health populations that draw doubt from providers can make it more difficult for patients to quit. This, in turn, can inhibit clinicians’ success in treating behavioral health populations for other illnesses, something providers need to be cognizant of [81]. From the most widespread barriers reported, there is a clear need for greater provider tobacco treatment education/training to increase the delivery of effective evidence-based interventions and to address provider attitudes that can inhibit cessation intervention efforts across care settings.

This study also adds to the literature by having identified that the number of reported barriers varied greatly across different health care center types, with the extremes being that standalone substance use treatment centers providers reported eight key barriers versus the single barrier reported by providers at substance use treatment programs within LMHAs. The abundance of barriers that were reported for standalone substance use treatment centers may reflect the limited funding these centers have [82]. Furthermore, it may also be suggestive of the low number of employees in these settings and high turnover rates, which could create frenetic workplace paces with prominent competing demands [83]. Despite this, however, the percentage of standalone substance use treatment centers that mandated screenings was similar to that of other health care settings. This suggests that such mandates are possible in all health care settings, including the least resourced ones, though there may be a need for greater (or more frequently refreshed) support provision in these settings (e.g., more training, Certified Tobacco Treatment Specialists, TUA templates [84,85]) relative to others to address the myriad perceived barriers. 

There are a few key practices that can help to address perceived barriers to tobacco use disorder care and support providers to provide tobacco use screenings that may increase their consistent use, namely, programming a hard stop in the EHR that requires the input of patients’ tobacco use status, training providers to screen for tobacco use, and making a TUA template available to providers [24,84,86,87,88]. In the present study, mandated tobacco screenings were most common in health care settings where hard stops in EHRs for entry of tobacco use status were used than in settings in which they were not; likewise, screening was marginally more common in centers where provider tobacco training was readily available than in settings where training was not offered. Within the context of tobacco use, hard stops in EHRs are under-addressed in clinical practice guidelines that outline steps providers can take to treat tobacco dependence [34]. Moreover, multiple studies have demonstrated the importance of EHR systems in streamlining intervention by facilitating screenings, a necessary first step to better understanding patients’ risk for tobacco-related disease [89,90,91]. However, barriers to the effectiveness of this key practice include that EHRs are not pervasive within all health care centers, not all EHRs have or easily allow modification to add a hard stop for tobacco use status, and implementation/modification of EHRs can be costly, which may be prohibitive for low-resource settings [47,92,93,94]. Additionally, hard stops/alerts in EHRs are overridden by providers at concerningly high rates, defeating their purpose [95]. Thus, treatment centers may need to find creative ways to ensure provider compliance with tobacco screening, potentially through other clinician decision supports when these circumstances exist. Similarly, access to tobacco training was marginally reported more frequently in centers that mandated tobacco screenings and has often been cited as a solution for improving tobacco cessation care [27,37,39]. Indeed, tobacco training has been recognized as a driving force in changing health care providers’ knowledge and attitudes, heightening its benefit in any setting [39]. Moreover, unlike hard stops in the EHRs, training can happen in all health care settings, including those with paper patient records that preclude electronic hard stops, and is recommended to improve care delivery regardless of whether specific health care practices are mandated or not [34,96].

The current study facilitated the ability to examine how practices supportive of tobacco screening varied by health care center type. For example, substance use treatment programs within LMHAs had the lowest reported presence of hard stops or tobacco screening training but did not differ from other center types in mandating tobacco screenings. Although this is suppositional, the relative lack of trainings and hard stops within substance use treatment centers in LMHAs may have been compensated for by important tobacco cessation care practices in which they excelled in comparison with other center types, including having a Certified Tobacco Treatment Specialist on-site (60% vs. 26–33.3%) and TUA template availability (80% vs. 50% of standalone substance use treatment centers and 43.8% of global LMHAs). Additionally, TUA template provision was marginally lower in global LMHAs and standalone substance use treatment centers relative to FQHCs and substance use treatment programs in LMHAs (43.8% and 50% vs. 88.9% and 80%), again revealing opportunities for growth in specific health care settings. Sharing templates within systems seems one efficient way to address this difference, at least between affiliated LMHA stakeholders, whereas other centers might use or adapt templates available online (for example, see here or, with lung cancer screening eligibility, here). Finally, although it was not a statistically significant difference, tobacco training availability also appeared lower in standalone and LMHA substance use treatment center settings compared with global LMHAs (42% and 40% vs. 62.5%). Access to training may remain limited in these types of settings due to budget constraints; however, efforts could be made to better disseminate information about free online trainings, such as those offered by the American Lung Association or the Smoking Cessation Leadership Center, to providers within them, providing avenues for future knowledge gain [82]. 

Overall, results from this study indicate that centers that mandated tobacco use screening used some, but not all, key practices to increase provider compliance with this policy. The current data are cross-sectional; therefore, the results cannot shed light on if key practices led to screening mandates or if screening mandates were accompanied by key practices to ensure their implementation. While it stands to reason that the provision of tools to increase the use and ease of screening conduct would increase the chances of its routine use in practice, more research is needed on the relative contribution of key practices to improving tobacco screening of patients in various settings to inform specific recommendations. Thus, until there is more work, the value of key practices that were not significantly or independently associated with mandated screenings should not be discounted as unimportant. Additionally, and although these were not the specific focus of this study, tobacco-free workplace policies have been found to help reduce tobacco use by enhancing physical cues in treatment center environments to facilitate a quit attempt; likewise, the presence of Certified Tobacco Treatment Specialists has been linked with evidence-based intervention provision [84,85,86,97]. Unfortunately, tobacco-free workplace policy implementation was reported at (marginally significant) lower rates in substance use treatment centers, both those in LMHAs and standalone centers, compared with the other types of surveyed centers, revealing a need to intervene to increase their uptake in these settings. Further, although it was again not statistically significant, the presence of Certified Tobacco Treatment Specialists was more common within LMHAs’ substance use treatment centers than in other surveyed health care settings. Thus, the current study indicated opportunities for implementing several best practices within each center type, which together may help to change provider and patient norms about tobacco use and, ultimately, to facilitate screening and intervention for tobacco use disorder. 

There are several limitations of relevance in this study. One limitation that may affect the generalizability of results is the limited pool of health care centers that were sampled, coupled with the reduction in sample size to include only direct service provider respondents. Moreover, comparability with the 2016 Marynak et al. study results is questionable given that their sample size was 361 mental health centers in Texas, even though that mental health centers in Texas are governed by only 39 LMHAs across the state [24,57]. It is possible that affiliated centers were not nested within LMHAs in the prior study, whereas our approach was to survey at the LMHA level given known consistencies in policies within those units. Nevertheless, better surveillance over time in all health care center types providing behavioral health care in Texas is needed to better understand the impacts of ongoing tobacco control efforts and to identify future needs to enhance the impacts of tobacco control spending. One strength of the current work is the inclusion of FQHCs in our sampling, a setting in which low-income and behavioral populations commonly receive tobacco care—especially in rural settings [56,98]. FQHCs are not typically included in surveys that reflect tobacco use disorder care for behavioral health care patients but may represent important stakeholders to engage to meet the tobacco use care needs of Texans [24,67]. Another limitation of this study included that although survey respondents were asked to describe policies and practices within the center overall, endorsements represented only respondents’ perceptions of what factors limited their center’s provision of tobacco screening and intervention and may not have reflected the position of all providers in the center. Moreover, in some cases, it may be that providers thought that patients were not interested in quitting tobacco but that that was a consequence of never having been offered advice or assistance from the provider to quit. Additionally, it is possible that respondents unknowingly provided nonrepresentative answers (e.g., a newer employee might be unaware if tobacco training was offered before they were onboarded). Future studies may improve upon this design by collecting data about employment duration or mandating that respondents have a certain duration of employment to participate. Furthermore, it might also be useful to assess whether providers actively utilized the key resources (e.g., tobacco use templates that may have been provided, attended tobacco trainings that may have been offered) that may have impacted tobacco use screening or interventions. Future work should include queries on the use of resources to improve the accuracy of reported findings, particularly when individual provider practices are assessed. Lastly, this study cannot determine causality due to its cross-sectional nature, limiting the extent to which conclusions can be drawn.

## 5. Conclusions

The findings of this study have important implications for health care centers, with particular emphasis on those treating populations with behavioral health needs; the disproportionate use of tobacco in this group is a concerning problem that can be addressed by increasing the occurrence of tobacco screenings as a tool to facilitate evidence-based cessation intervention [15,17]. This study demonstrates that mandated tobacco screenings were most common in health care settings where hard stops in EHRs were present and marginally more common when provider tobacco screening training was readily available. Furthermore, lack of knowledge or confidence in their abilities, time, and community resources to refer patients; importance of treating competing diagnoses; and belief that patients do not comply with treatment recommendations were all identified as the most widespread barriers to providers conducting tobacco screening and intervention, with the different types of health care centers ranging widely in the number of key barriers that were reported. Notably, most of the reported barriers were rooted in either providers’ perceived importance of treating tobacco use or their attitudes associated with behavioral health patients’ quitting; these can be feasibly overcome through greater provider education. The current work suggests some key strategies for screening behavioral health care patients more consistently for tobacco use. Future research could focus on how tobacco screening practices affect cessation services offered, potentially by health care center type, as that information may prove to be instrumental in further reducing research-to-practice gaps in tobacco use disorder care.

## Figures and Tables

**Table 1:** Health Care Center Characteristics and Tobacco Use Screening Practices (*n* = 80).

Center Characteristics and Practices	All Centers (*n* = 80)	FQHC (*n* = 9)	Global LMHA (*n* = 16)	LMHA SUT (*n* = 5)	SUTC (*n* = 50)	*X* ^2^	*p*-Value
	% (*n*)						
# of unique patients/yearly						38.701	<0.001
50–200	30.77 (24)	25.00 (2)	0.00 (0)	20.00 (1)	42.00 (21)		
201–1000	44.87 (35)	37.50 (3)	20.00 (3)	40.00 (2)	54.00 (27)		
>1000	24.36 (19)	37.50 (3)	80.00 (12)	40.00 (2)	4.00 (2)		
# of full-time employees						34.254	<0.001
1–50	61.25 (49)	33.33 (3)	6.25 (1)	60.00 (3)	84.00 (42)		
>50	38.75 (31)	66.67 (6)	93.75 (15)	40.00 (2)	16.00 (8)		
Has ≥ 1 CTTS						2.583	0.442
Yes	30.00 (24)	33.33 (3)	31.25 (5)	60.00 (3)	26.00 (13)		
No/I do not know	70.00 (56)	66.67 (6)	68.75 (11)	40.00 (2)	74.00 (37)		
Has a comprehensive TFW policy						7.228	0.065
Yes	50.00 (40)	77.78 (7)	68.75 (11)	40.00 (2)	40.00 (20)		
No	50.00 (40)	22.22 (2)	31.25 (5)	60.00 (3)	60.00 (30)		
Mandates tobacco use screening						0.043	1.000
Yes	80.00 (64)	77.78 (7)	81.25 (13)	80.00 (4)	80.00 (40)		
No/I do not know	20.00 (16)	22.22 (2)	18.75 (3)	20.00 (1)	20.00 (10)		
TUA template availability						6.762	0.077
Yes	55.00 (44)	88.89 (8)	43.75 (7)	80.00 (4)	50.00 (25)		
No/I do not know	45.00 (36)	11.11 (1)	56.25 (9)	20.00 (1)	50.00 (25)		
EHR hard stop/documentation required						1.506	0.693
Yes	65.00 (52)	66.67 (6)	68.75 (11)	40.00 (2)	66.00 (33)		
No/I do not know	35.00 (28)	33.33 (3)	31.25 (5)	60.00 (3)	34.00 (17)		
Received or offered training to screen						2.153	0.569
Yes	46.25 (37)	44.44 (4)	62.50 (10)	40.00 (2)	42.00 (21)		
No/I do not know	53.75 (43)	55.56 (5)	37.50 (6)	60.00 (3)	58.00 (29)		

Note: FQHC = Federally Qualified Health Center; LMHA = Local Mental Health Authority (mental health services); LMHA SUT = Local Mental Health Authority Substance Use Treatment program; SUTC = standalone Substance Use Treatment Center; CTTS = Certified Tobacco Treatment Specialist; TFW = Tobacco-free Workplace.

**Table 2:** The Association of Health Care Center Tobacco Use Screening Mandates in Relation to Health Care Center Practices/Resources, Controlling for Health Care Center Type and Comprehensive Tobacco-Free Workplace Policy Presence (*n* = 80).

	Estimate	SE	OR	95% CI		*p*-Value
Key Independent Variables						
TUA template availability	0.482	0.705	1.620	0.407	6.446	0.494
EHR hard stop/documentation required	1.396	0.679	4.039	1.067	15.288	0.040
Received or offered training to screen	1.584	0.871	4.874	0.885	26.848	0.069
Control Variables						
FQHC	−0.842	1.086	0.431	0.051	3.621	0.438
Global LMHA	−0.448	0.869	0.639	0.116	3.508	0.606
LMHA SUT	0.477	1.359	1.612	0.112	23.127	0.725
Has comprehensive TFW policy	0.900	0.753	2.458	0.562	10.764	0.233

Note: Intercept not shown. TUA = Tobacco Use Assessment; EHR = Electronic Health Record; SE = Standard Error; OR = Odds Ratio; CI = Confidence Interval; FQHC = Federally Qualified Health Center; Global LMHA = Local Mental Health Authority (mental health services); LMHA SUT = Local Mental Health Authority Substance Use Treatment Program; SUTC = standalone Substance Use Treatment Center; CTTS = Certified Tobacco Treatment Specialist; TFW = Tobacco-free Workplace. For Key Independent Variables, the reference group is “no/I do not know”; for health care center type, SUTC is the reference group; for comprehensive TFW policy, no comprehensive TFW policy is the reference group.

## Data Availability

The data presented in this study are available on request from the corresponding author. The data are not publicly available due to funder restrictions and because outcome papers are still being reported from this dataset.
